# Changes in Aniseikonia in Patients with Idiopathic Epiretinal Membrane Treated with Pars Plana Vitrectomy: A Meta-Analysis

**DOI:** 10.3390/jcm15114170

**Published:** 2026-05-28

**Authors:** Gyudeok Hwang, Mee Yon Lee, Jeong Ah Shin, Min Young Lim, Young Seong Yang, Jayoung Ahn, Heesung Sohn, Joonhong Sohn

**Affiliations:** 1 Gimpo Hangil Eye Center, Gimpo-si 10110, Republic of Korea; mehwang78@naver.com (G.H.); my0514lim@gmail.com (M.Y.L.); ysyang1318@naver.com (Y.S.Y.); 2 Department of Ophthalmology, Uijeoungbu St. Mary’s Hospital, College of Medicine, The Catholic University of Korea, Uijeoungbu 11765, Republic of Korea; deenie79@gmail.com; 3 Department of Ophthalmology, Yeouido St. Mary’s Hospital, College of Medicine, The Catholic University of Korea, Seoul 07345, Republic of Korea; nole0710@gmail.com; 4 Department of Ophthalmology, Hangil Eye Hospital, Incheon 21388, Republic of Korea; bluekami01@naver.com; 5 Department of Information Technology Development, Hangil Eye Hospital, Incheon 21388, Republic of Korea; surasurato@naver.com

**Keywords:** aniseikonia, epiretinal membrane, meta-analysis, pars plana vitrectomy

## Abstract

**Objectives:** This study aimed to assess longitudinal changes in aniseikonia following pars plana vitrectomy (PPV) in patients with idiopathic epiretinal membrane (ERM). **Methods:** This meta-analysis was conducted in accordance with the PRISMA guidelines. The PubMed, EMBASE, and Cochrane Library databases were searched from inception to March 2024 to identify studies evaluating aniseikonia outcomes after PPV for idiopathic ERM. Studies involving secondary ERM or those lacking aniseikonia assessments were excluded. Correlations between aniseikonia and best-corrected visual acuity (BCVA) and central macular thickness (CMT) were analyzed. **Results:** Ten studies comprising 456 eyes were included. PPV was associated with a statistically significant reduction in aniseikonia at all evaluated postoperative time points, including 1 month (−1.09%, *p* = 0.04), 3 months (−0.81%, *p* = 0.02), 6 months (−0.89%, *p* = 0.002), and 12 months (−1.13%, *p* < 0.001). Meta-analysis of outcomes at the final follow-up of each study also demonstrated a significant overall improvement in aniseikonia (−1.09%, *p* < 0.001). Subgroup analysis revealed a statistically significant improvement only in studies with long-term follow-up (≥12 months), whereas studies with short-term follow-up (≤6 months) did not show significant changes. No significant correlations were found between changes in aniseikonia and changes in BCVA, CMT, or other structural parameters. **Conclusions:** Aniseikonia showed a statistically significant but modest reduction after PPV for idiopathic ERM. However, the magnitude and consistency of improvement varied across studies, and its clinical significance remains uncertain. Improvements appeared gradual and more evident with longer follow-up, suggesting that longer-term follow-up may be necessary to adequately assess postoperative changes in aniseikonia.

## 1. Introduction

Epiretinal membrane (ERM) is a common macular disorder that can lead to visual distortions, including aniseikonia, which is characterized by a perceived difference in image size between the two eyes [1]. Aniseikonia encompasses both micropsia, in which objects appear smaller than their actual size, and macropsia, in which objects appear larger than they actually are. When mechanical forces applied to the retina stretch the photoreceptors, a given image activates a reduced number of receptors, resulting in the perception of a smaller image (micropsia). In contrast, the compression of photoreceptors leads to the activation of a greater number of receptors in the same image, producing the perception of an enlarged image (macropsia) [2]. Patients with ERM predominantly experience macropsia, although micropsia has also been reported [1].

Several methods are available for measuring aniseikonia, including the New Aniseikonia Test (NAT; Handaya Co., Ltd., Tokyo, Japan), Aniseikonia Inspector (AI; Optical Diagnostics, Culemborg, The Netherlands), Brecher test, Miles test, and Space Eikonometer. Among these, the NAT and AI are commercially available and have well-established validity and reliability [1]. The NAT is administered using a booklet containing adjacent calibrated pairs of red and green half-circular targets of varying sizes. Using red/green analytical filters for dissociation, one half-circle is perceived by the right eye and the other by the left eye, enabling the assessment of perceived image size differences between the two eyes [3]. The AI is a computerized test that quantifies aniseikonia by presenting red/green rectangular targets for direct size comparison. Patients indicate which target appears larger, and their responses are used to generate a psychometric function that reflects perceived image size differences [4].

Pars plana vitrectomy (PPV) is the standard surgical treatment for ERM; however, its effectiveness in improving aniseikonia remains controversial. Although some studies have reported no significant improvement in aniseikonia after PPV, others have demonstrated significant changes. For example, Fukuyama et al. [5] reported no significant improvements in aniseikonia 6 months after PPV in patients with ERM when idiopathic and secondary ERM were grouped together. Similarly, Ichikawa et al. [6] and Nakashizuka et al. [7] observed no statistically significant changes in aniseikonia 3 and 12 months after PPV, respectively, in studies that included both idiopathic and secondary ERM. Okamoto et al. [8] also reported minimal improvements in aniseikonia 6 months after PPV, suggesting that heterogeneity in the pathogenesis of ERM may influence outcomes. In contrast, Kim et al. [9] found significant improvements in aniseikonia only in cases of idiopathic ERM, whereas cases of secondary ERM, such as those associated with retinal breaks, showed no meaningful changes 6 months after PPV. These findings highlight the importance of focusing specifically on idiopathic ERM when analyzing aniseikonia outcomes because pooling data from heterogeneous ERM populations may obscure the therapeutic effects of PPV.

Idiopathic ERM is primarily associated with proliferation of glial cells, including Müller cells, and typically presents as a uniform circular global attachment to the macula [10,11]. This even distribution facilitates a more stable macular microstructure after surgery, likely enabling photoreceptor realignment and improving visual symmetry, which contributes to aniseikonia reduction. Secondary ERM often arises from retinal pigment epithelial cells and exhibits irregular finger-like projections with focal attachment patterns [12]. This morphology may account for the limited improvement observed in secondary ERM, as irregularities introduce persistent structural disruptions. Additionally, idiopathic ERM tends to develop in older individuals with gradual macular changes, whereas secondary ERM is often associated with traumatic or inflammatory events in younger patients [11]. These conditions introduce chronic retinal damage, edema, or scarring, further limiting postoperative functional recovery.

This meta-analysis aimed to determine whether PPV leads to statistically significant improvements in aniseikonia in idiopathic ERM and to examine the trajectory of these changes over time. By analyzing data exclusively from studies including idiopathic ERM, this study aimed to clarify the effectiveness of PPV in improving aniseikonia and to define an appropriate duration for postoperative monitoring.

## 2. Materials and Methods

### 2.1. Search Strategy and Study Selection

A systematic search was conducted using PubMed, EMBASE, and the Cochrane Library from inception to March 2024. This study aimed to identify studies that have evaluated the effect of PPV on aniseikonia in patients with idiopathic ERM. Search terms included combinations of Medical Subject Headings, Embase subject headings (Emtree), and keywords such as “epiretinal membrane,” “macular pucker,” “vitrectomy,” “aniseikonia,” and “metamorphopsia.” The full search terms were ((“Epiretinal Membrane”[MeSH] OR “Epiretinal Membranes”[TW] OR “Membrane, Epiretinal”[TW] OR “Cellophane Maculopathy”[TW] OR “Cellophane Maculopathies”[TW] OR “Maculopathy, Cellophane”[TW] OR “Premacular Fibrosis”[TW] OR “Fibrosis, Premacular”[TW] OR “Premacular Fibroses”[TW] OR “Macular Puckers”[TW] OR “Macular Pucker”[TW] OR “Pucker, Macular”[TW] OR “Preretinal Membrane”[TW] OR “Membrane, Preretinal”[TW] OR “Preretinal Membranes”[TW] OR “Epimacular Membrane”[TW] OR “Epimacular Membranes”[TW] OR “Membrane, Epimacular”[TW] OR “Surface-Wrinkling Retinopathy”[TW] OR “Retinopathy, Surface-Wrinkling”[TW] OR “Surface Wrinkling Retinopathy”[TW] OR “Surface-Wrinkling Retinopathies”[TW] OR “Preretinal Macular Fibrosis”[TW] OR “Fibrosis, Preretinal Macular”[TW] OR “Macular Fibrosis, Preretinal”[TW] OR “Preretinal Macular Fibroses”[TW]) OR ((Epiretinal membrane) OR (Epiretinal membranes) OR (Macular Puckers) OR (Macular Pucker))) AND (((“Aniseikonia”[MeSH] OR “Aniseikonia”[TW]) OR (Aniseikonia)) OR ((“Metamorphopsia”[TW] OR “Metamorphopsias”[TW]) OR (Metamorphopsia OR Metamorphopsias))).

The inclusion criteria were as follows: (1) diagnosis of idiopathic ERM; (2) treatment with PPV; and (3) reporting of outcomes related to aniseikonia. The exclusion criteria were: (1) studies involving secondary ERM or where idiopathic and secondary ERM were not clearly differentiated, (2) studies not including PPV as an intervention, and (3) studies that did not assess aniseikonia.

**Figure 1:**
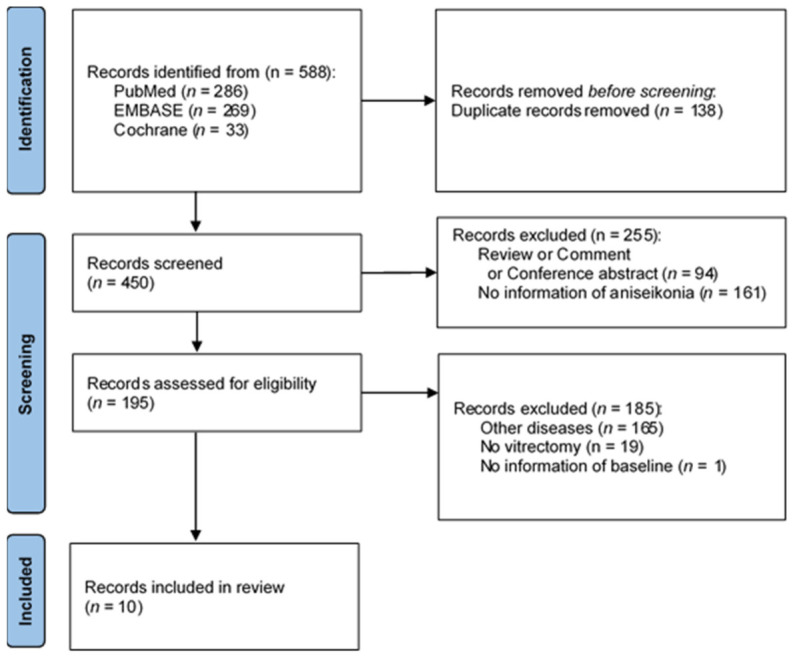
Flow diagram of study selection.

Of the 588 articles initially identified, duplicates were removed and titles and abstracts were screened independently by two investigators, resulting in 433 potentially relevant studies. Full-text reviews further narrowed the selection to 10 eligible studies that focused exclusively on idiopathic ERM (Figure 1). Any discrepancies in the study selection were resolved through discussion.

We have retrospectively registered this study in a public registry, PROSPERO. The registration record number is 1336412.

### 2.2. Data Extraction

The data extracted from each study are presented in Table 1. The key attributes included the first author, publication year, country of study, study design, number of eyes included, follow-up duration, aniseikonia measurement methods, and outcomes assessed. Additional methodological details, such as the gauge of PPV instrumentation and analyses of macular configurations, including central macular thickness (CMT), foveal avascular zone (FAZ), ellipsoid zone disruption (EZD), ectopic inner foveal layer (EIFL), inner retinal layers including the inner nuclear layer (INL), and ganglion cell-inner plexiform layer (GCIPL), were also extracted.

The results were categorized based on the statistical significance of aniseikonia changes, PPV gauge used, and follow-up duration. For studies reporting best-corrected visual acuity (BCVA) in the Snellen format, values were converted into logarithm of the minimum angle of resolution (logMAR) using the formula (logMAR = −1 × log_10_ [Snellen fraction]). Numerical data presented only in figure format were extracted using a Web Plot Digitizer (version 4.4; Automeris LLC, Austin, TX, USA). The term “end of the study” refers to the completion of each trial or the final follow-up point.

### 2.3. Quality Assessment

**Figure 2:**
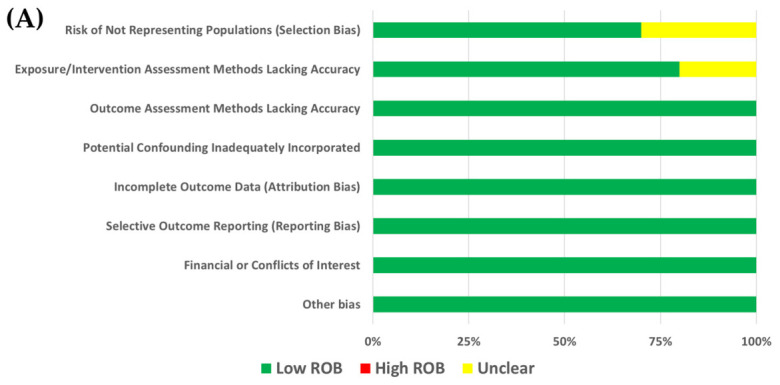

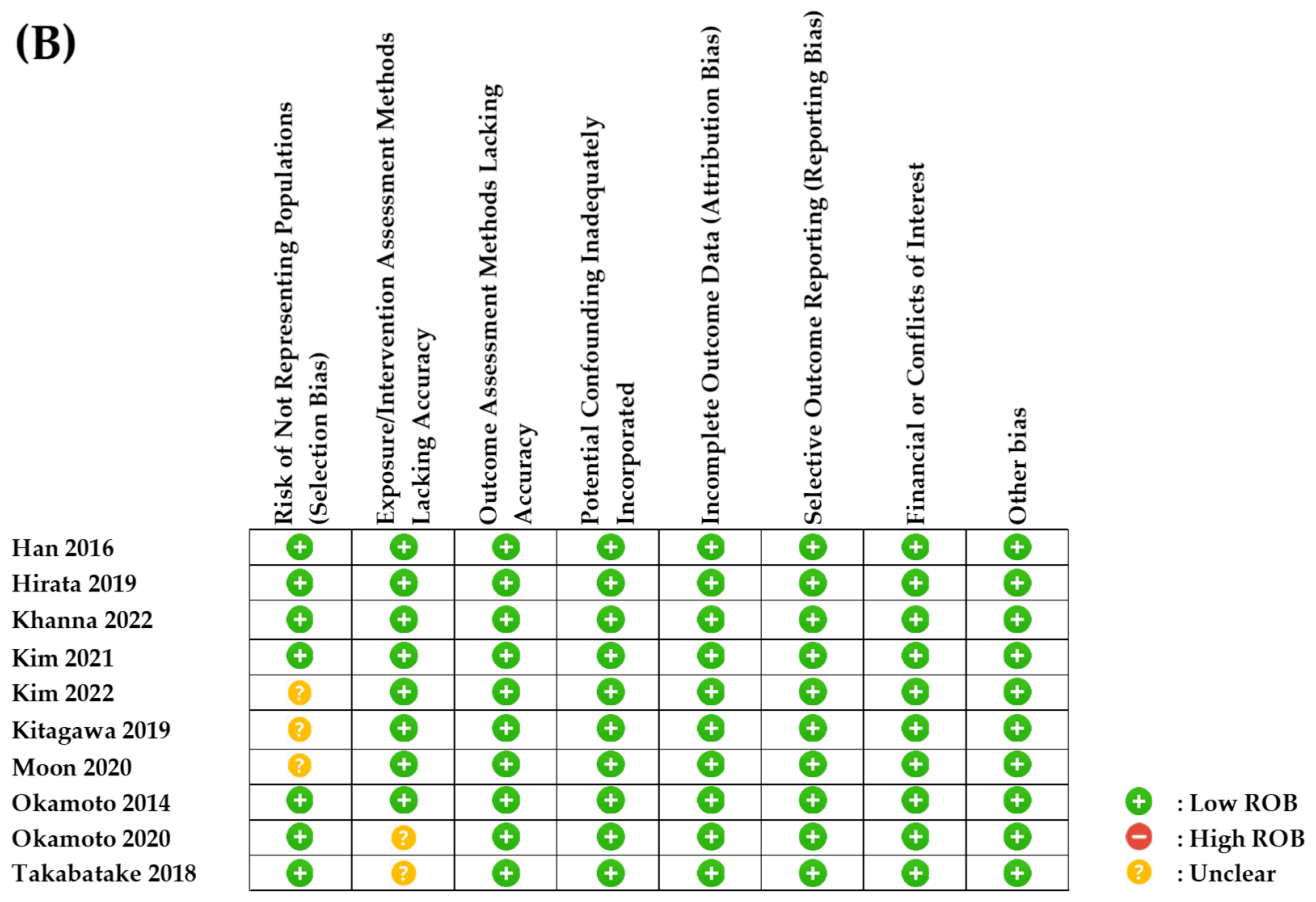
Risk of bias ratings in the included studies [9,13,14,15,16,17,18,19,20,21]. (**A**) Risk of bias graph showing the proportion of studies with low (green), high (red), and unclear (yellow) risk across each domain. (**B**) Risk of bias summary showing the risk of bias for each included study. ROB, risk of bias.

The Newcastle–Ottawa Scale was used to assess the quality of the included studies. Each study was evaluated for participant selection, cohort comparability, and outcomes. Studies that lacked comparative designs or did not clearly report participant retention rates were rated as having an unclear risk in specific Newcastle–Ottawa Scale domains. Two investigators independently performed quality assessment and resolved disagreements by consensus. A summary of the quality assessment is shown in Figure 2.

### 2.4. Statistical Analyses

The statistical analyses adhered to the Preferred Reporting Items for Systematic Reviews and Meta-Analyses (PRISMA) guidelines. Data were analyzed using RevMan version 5.3 (Cochrane, London, UK). Continuous outcomes were synthesized using a random-effects model based on the inverse variance method to calculate pooled mean differences with 95% confidence intervals. The random-effects model was implemented to account for between-study variability. Preoperative and postoperative mean values, standard deviations, and sample sizes reported in each study were directly entered into RevMan for the analysis of aniseikonia outcomes. Heterogeneity was quantified using the I^2^ statistic, with low, moderate, and severe heterogeneity defined as I^2^ values of ≤25%, 25–75%, and >75%, respectively.

**Figure 3:**
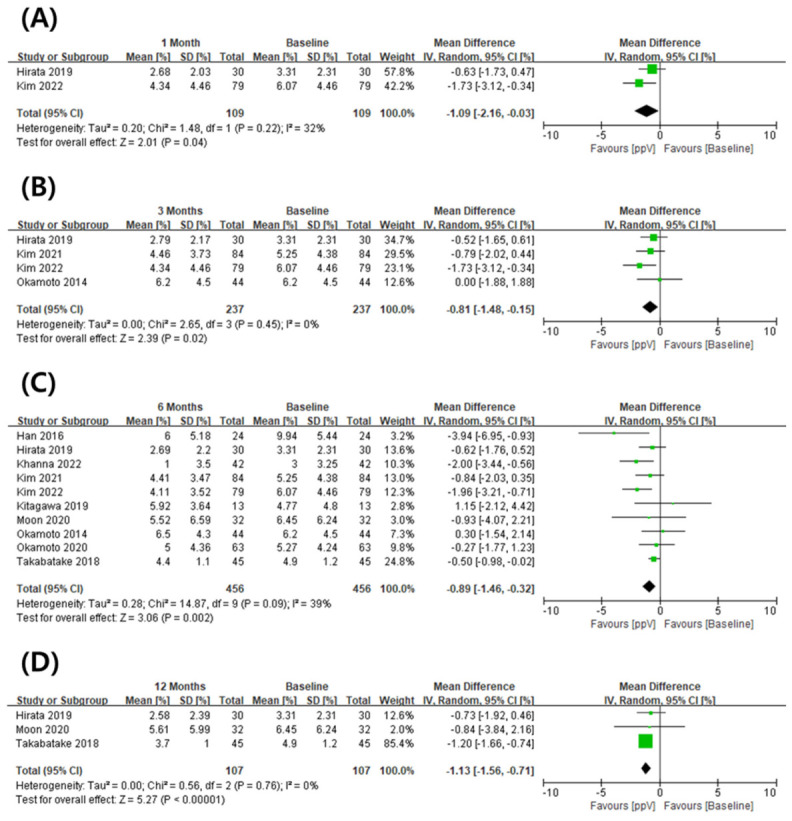
Forest plots of longitudinal changes in the aniseikonia (**A**) at 1 months, (**B**) at 3 months, (**C**) 6 months, and (**D**) 12 months after pars plana vitrectomy (PPV). Analyses were performed using a random-effects model based on the inverse variance method [9,13,14,15,16,17,18,19,20,21]. PPV, pars plana vitrectomy; SD, standard deviation; IV, the inverse variance method; Random, a random-effects model; CI, confidence interval.

**Figure 4:**
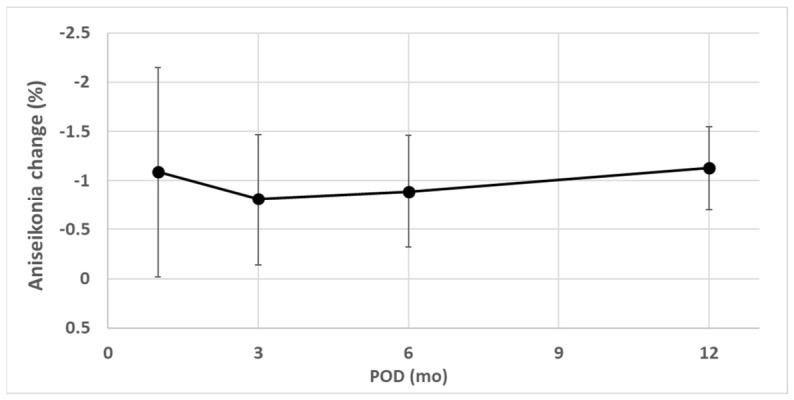
Longitudinal changes in the aniseikonia after pars plana vitrectomy. Analyses were performed using a random-effects model based on the inverse variance method. Error bars represent lower and upper limits of 95% confidence intervals. POD, postoperative day; mo, months.

**Figure 5:**
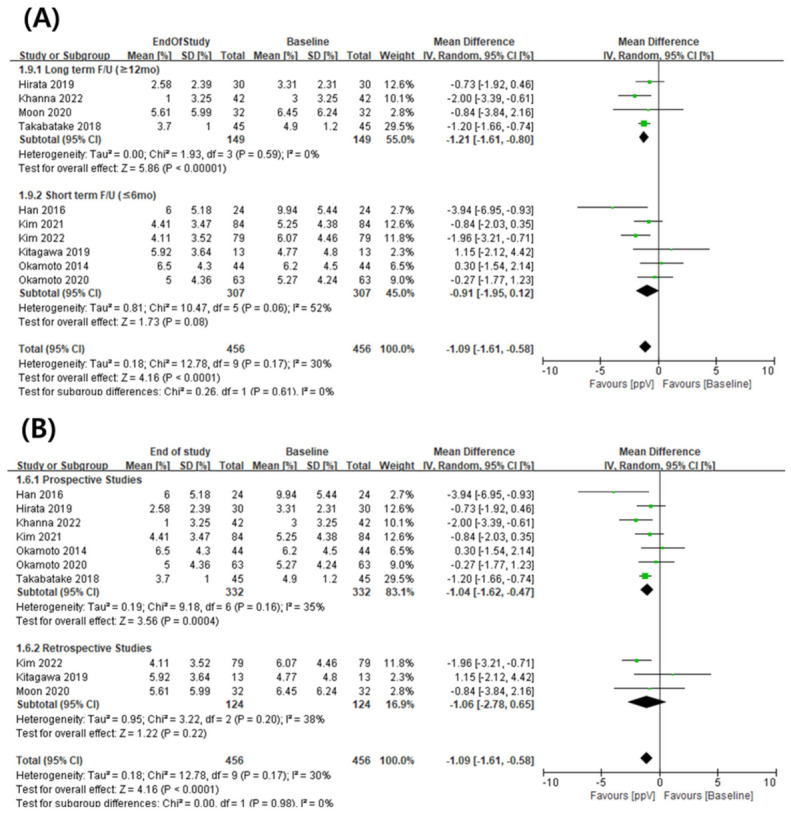
Forest plots of changes in the aniseikonia at the final follow-up after pars plana vitrectomy (PPV) with subgroup analyses according to (**A**) follow-up period and (**B**) study design. Analyses were performed using a random-effects model based on the inverse variance method [9,13,14,15,16,17,18,19,20,21]. PPV, pars plana vitrectomy; SD, standard deviation; IV, the inverse variance method; Random, a random-effects model; CI, confidence interval; F/U, follow-up; mo, months.

**Figure 6:**
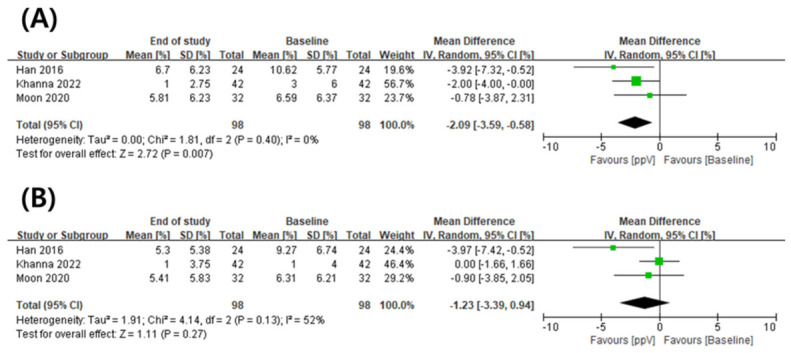
Forest plots of pooled mean differences in aniseikonia at the final follow-up after pars plana vitrectomy (PPV), showing (**A**) vertical aniseikonia and (**B**) horizontal aniseikonia. Analyses were performed using a random-effects model based on the inverse variance method [13,15,18]. PPV, pars plana vitrectomy; SD, standard deviation; IV, the inverse variance method; Random, a random-effects model; CI, confidence interval.

Subgroup analyses were performed to evaluate the impact of the follow-up duration, study design (prospective vs. retrospective), and other methodological differences on aniseikonia outcomes. Forest plots were generated to illustrate the pooled effect sizes and subgroup analyses (Figure 3, Figure 4, Figure 5 and Figure 6). Correlation analyses between changes in aniseikonia and structural/functional parameters, such as BCVA and CMT, were performed using Spearman’s rank correlation. All statistical tests were two-sided, and the threshold for statistical significance was set to *p* < 0.05.

## 3. Results

### 3.1. Characteristics of the Included Studies

Table 1 summarizes the characteristics of the ten studies [9,13,14,15,16,17,18,19,20,21] included in this meta-analysis, including both prospective (n = 7) and retrospective (n = 3) studies. A total of 456 eyes were analyzed across the included studies, with the number of eyes per study ranging from 13 to 84. The follow-up durations were 6 months (n = 6), 12 months (n = 3), and 24 months (n = 1). The included studies were conducted in Korea, Japan, and France, with publication years ranging from 2014 to 2022. The studies employed different tools to measure aniseikonia, with one study using AI and nine using NAT.

The study selection process began with 588 records retrieved from the PubMed, EMBASE, and Cochrane databases, which, after screening and applying the eligibility criteria, resulted in the final inclusion of 10 studies (Figure 1). These studies focused on idiopathic ERM treated with PPV and evaluated postoperative changes in aniseikonia. Notably, five studies reported significant improvements in aniseikonia following PPV, whereas the other five studies did not observe statistically significant changes.

### 3.2. Quality Assessment

Quality assessment (Figure 2) indicated that the overall risk of bias was generally low to moderate, with several domains being unclear across the included studies. Five studies had an unclear risk of bias, and the other five had a low risk of bias. The quality of each study was evaluated using the Newcastle–Ottawa Scale, which assesses study selection, comparability, and outcome assessment.

### 3.3. Changes in Aniseikonia

The meta-analysis found a statistically significant overall improvement in aniseikonia following PPV in patients with idiopathic ERM. Figure 3 and Figure 4 illustrate the longitudinal progression of aniseikonia across different follow-up periods. At 1 month postoperatively, the pooled effect size was −1.09% (95% confidence interval [CI], −2.16% to −0.03%; *p* = 0.04). This improvement was slightly attenuated at 3 months (−0.81%; 95% CI, −1.48% to −0.15%; *p* = 0.02), followed by a sustained and significant improvement at 6 months (−0.89%; 95% CI, −1.46% to −0.32%; *p* = 0.002). The magnitude of improvement further increased at 12 months, reaching −1.13% (95% CI, −1.56% to −0.71%; *p* < 0.001).

Although the final follow-up time points varied among the 10 included studies (6 months in six studies, 12 months in three studies, and 24 months in one study), we performed a meta-analysis using pooled aniseikonia outcomes measured at the final follow-up point of each study. This pooled analysis demonstrated a significant overall improvement in aniseikonia, with a mean difference of −1.09% (95% CI, −1.61% to −0.58%; *p* < 0.001) at the end of the study (Figure 5).

The effect of the follow-up duration on measured aniseikonia improvements was evaluated by comparing studies with short-term (≤6 months; n = 6 studies, 307 eyes) and long-term (≥12 months; n = 4 studies, 149 eyes) follow-ups. Significant improvements in aniseikonia were primarily observed in the long-term follow-up subgroup, with a pooled mean difference of −1.21% (95% CI, −1.61% to −0.80%; *p* < 0.001). These findings suggest that the beneficial effects of PPV on aniseikonia may become more evident over extended follow-up periods (Figure 5A). In addition, subgroup analyses according to the study design revealed that prospective studies, which are generally less susceptible to bias, showed a significant reduction in aniseikonia, whereas retrospective studies did not demonstrate a statistically significant change (Figure 5B). Moreover, the subgroup analysis of vertical versus horizontal aniseikonia changes revealed that only vertical aniseikonia showed a statistically significant improvement, whereas horizontal aniseikonia did not change significantly (Figure 6).

Heterogeneity across studies ranged from 0% to 52%, indicating low to moderate heterogeneity. This suggests relatively consistent effects across the included studies, demonstrating the robustness of the pooled analysis. In the analysis of longitudinal changes in the aniseikonia, the I^2^ value was 32% at the 1st month, 0% at the 3rd month, 39% at the 6th month, and 0% at the 12th month (Figure 3). In the subgroup analysis according to study designs, the I^2^ value was similar at 35% in the prospective study group and 38% in the retrospective study group (Figure 5B). On the other hand, in the subgroup analysis according to follow-up period, the I^2^ value was low at 0%, but the I^2^ value was slightly higher at 52% in the short-term follow-up group (Figure 5A).

**Figure 7:**
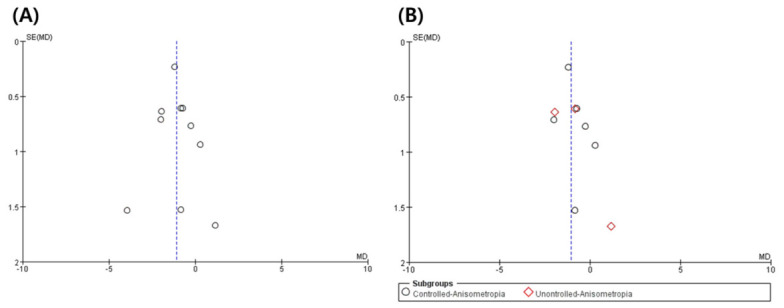
Funnel plot analyses assessing potential publication bias and methodological heterogeneity in studies evaluating postoperative changes in aniseikonia after pars plana vitrectomy for idiopathic epiretinal membrane. (**A**) Funnel plot including all studies. (**B**) Funnel plot excluding the study using the Aniseikonia Inspector and stratified according to anisometropia control status. SE(MD), standard error of the mean difference; MD, mean difference.

Publication bias was additionally assessed using funnel plot analyses (Figure 7). Mild asymmetry was observed in the overall funnel plot, but this asymmetry was substantially attenuated after exclusion of the study using the AI and subgroup stratification according to anisometropia control status. These findings suggest that methodological heterogeneity may have contributed more substantially to effect-size variability than publication bias itself. However, interpretation remained limited because of the small number of included studies.

### 3.4. Correlation Analyses of Aniseikonia Changes with Visual and Structural Parameters

Table 2 presents the results of correlation analyses among aniseikonia changes, BCVA, and CMT. There was no statistically significant correlation between changes in aniseikonia and changes in BCVA (correlation coefficient: 0.6371; *p* = 0.065) or CMT (correlation coefficient: 0.0599; *p* = 0.888). Furthermore, preoperative BCVA and CMT values did not significantly correlate with postoperative aniseikonia improvement, suggesting that preoperative retinal thickness and visual acuity may not be predictive of aniseikonia outcomes after PPV. Supplementary Figures S1 and S2 demonstrate that BCVA and CMT consistently improved across all subgroups, with no similar differentiation when grouped by study design or follow-up duration. This further supports the absence of a correlation between BCVA and CMT, indicating that improvements in aniseikonia may be driven by factors other than retinal thickness and visual acuity.

## 4. Discussion

This meta-analysis evaluated longitudinal changes in aniseikonia following PPV for idiopathic ERM and demonstrated a statistically significant but modest reduction over time. Notably, the improvement in aniseikonia appeared to be gradual and nonlinear, with more pronounced changes observed at longer follow-up periods. These findings highlight the importance of long-term follow-up when assessing functional outcomes after PPV. Nevertheless, the magnitude and consistency of this improvement varied across studies.

Because the pooled reduction in aniseikonia was approximately 1%, the clinical significance of this magnitude requires careful interpretation. Previous studies have reported that aniseikonia of 1–2% may be present in normal individuals, whereas levels exceeding 3% are associated with impaired binocular vision and levels above 5% may result in severely compromised binocular function [22]. In the present analysis, baseline aniseikonia in most included studies ranged from approximately 3% to 6%, suggesting that even modest reductions may shift patients toward a less symptomatic or subclinical range. In addition, recent evidence indicates that even small degrees of aniseikonia may affect stereoacuity [23], supporting the potential functional relevance of relatively small changes. Even a ~1% reduction may be meaningful for some patients given baseline values of 3–6%. Nevertheless, the clinical impact of a reduction of approximately 1% remains uncertain and should be interpreted with caution.

In this context, subgroup analysis based on study design showed that statistically significant improvements in aniseikonia were observed only in prospective studies, whereas retrospective studies did not demonstrate significant changes (Figure 5B). Given that prospective studies are generally less susceptible to bias, this finding suggests that study design may have influenced the observed effect and should be considered when interpreting the results.

Subgroup analyses based on follow-up duration provided further insight into the temporal pattern of gradual improvement. Statistically significant improvements in aniseikonia were observed only in studies with long-term follow-up (≥12 months), whereas studies with short-term follow-up (≤6 months) did not demonstrate significant changes (Figure 5A). These findings suggest that a minimum follow-up period of at least 12 months may be required to capture the full extent of aniseikonia improvement after PPV for idiopathic ERM. This delayed recovery pattern is consistent with previous reports indicating that, while metamorphopsia may improve relatively early after surgery, aniseikonia tends to recover more slowly and progressively over time [21]. However, these subgroup analyses should also be interpreted cautiously. The results may partly reflect differences in study design, baseline severity, or statistical power rather than a true delayed recovery effect.

The mechanisms underlying aniseikonia improvement after PPV are likely multifactorial and remain incompletely understood. Structural factors such as retinal displacement and photoreceptor realignment following membrane removal may play an important role. In addition, cortical adaptation may contribute to the progressive improvement observed over time, as the visual system adjusts to altered retinal input after surgery. These mechanisms may help explain the delayed and nonlinear recovery pattern identified in this study; however, the proposed pathophysiological mechanisms remain speculative and were not directly supported by the included data.

The present analysis also revealed a distinct improvement in vertical aniseikonia postoperatively, whereas horizontal aniseikonia did not show significant changes (Figure 6). This finding contrasts with the metamorphopsia outcomes reported in previous studies, where horizontal metamorphopsia showed statistically significant improvement following PPV in patients with ERM and vertical metamorphopsia did not demonstrate meaningful changes during a 12-month follow-up period [21,24,25]. Additionally, the study by Ichikawa et al. [6] suggested that aniseikonia could be influenced by retinal displacement in both the tangential and axial planes, potentially differentially affecting directional improvement in individual cases. Predominant tangential traction vectors in ERM may also contribute to directional differences in retinal displacement and postoperative visual perception changes. This directional discrepancy suggests that mechanisms underlying aniseikonia improvement are likely complex and cannot be fully explained by uniform structural recovery alone. Nevertheless, the conclusion regarding vertical versus horizontal aniseikonia may be premature given the limited number of available studies and the potential variability in measurement methodologies.

Interestingly, despite clear improvements in both BCVA and CMT, this meta-analysis did not identify significant correlations between changes in these parameters and changes in aniseikonia (Supplementary Figures S1 and S2, Table 2). Similarly, preoperative BCVA and CMT were not associated with postoperative changes in aniseikonia. These findings suggest that anatomical recovery and visual acuity improvement do not necessarily translate into proportional improvements in perceptual image size disparity. This dissociation highlights the complexity of aniseikonia and indicates that it may represent a distinct visual outcome that cannot be fully explained by conventional structural or functional metrics.

In line with the lack of correlation between aniseikonia and conventional parameters, several macular microstructural parameters, including INL thickness, GCIPL thickness, and EZD, showed significant improvements at the final follow-up (Supplementary Figure S3B–D). However, no statistically significant associations were found between changes in aniseikonia and changes in these parameters (Spearman’s rank correlation analysis; *p* = 0.167, 0.167, and 0.667, respectively). In addition, FAZ did not show a significant change over time (Supplementary Figure S3A), nor was it correlated with aniseikonia changes (Spearman’s rank correlation analysis; *p* = 0.333). These analyses were based on a limited number of studies (n = 3 for each parameter), which may have reduced the statistical power to detect potential associations. Taken together, these findings suggest that currently measurable structural parameters may not fully account for the mechanisms underlying aniseikonia improvement.

However, these analyses were conducted at the study level and are therefore subject to potential ecological bias and limited statistical power. Accordingly, these findings should be interpreted cautiously and considered exploratory.

Differences between idiopathic and secondary ERM may also contribute to variability in reported outcomes. Studies that did not differentiate between idiopathic and secondary ERM, such as those by Fukuyama et al. [5], Ichikawa et al. [6], Nakashizuka et al. [7], and Okamoto et al. [8], consistently reported no significant postoperative improvement in aniseikonia. Although these studies did not meet the inclusion criteria, their findings suggest that differences between idiopathic and secondary ERM may influence postoperative aniseikonia outcomes. In contrast, Kim et al. [9] demonstrated that significant improvements occurred only in cases of idiopathic ERM, whereas cases of secondary ERM, such as those associated with retinal breaks, showed no significant changes. This may be related to differences in underlying pathophysiology, as idiopathic ERM is typically associated with posterior vitreous detachment and relatively uniform traction, whereas secondary ERM may involve more complex and heterogeneous retinal changes [10,17,21]. However, the hypothesis that idiopathic ERM may permit more stable microstructural recovery than secondary ERM remains speculative and was not directly supported by the included data.

Despite these findings, the underlying mechanisms explaining why aniseikonia improves after ERM surgery remain incompletely understood. No specific macular configuration was identified as being consistently associated with changes in aniseikonia, and half of the included studies did not demonstrate statistically significant improvement, highlighting the variability and uncertainty of the observed effect. In addition, the inclusion of studies with varying methodological rigor, particularly those that did not adequately control for anisometropia, may have influenced the reliability of the pooled estimates. As such, the results of this meta-analysis should be interpreted with caution and considered exploratory.

From a clinical perspective, these findings suggest that improvements in aniseikonia may evolve over time and may not be fully apparent within the early postoperative period. Therefore, clinicians should consider long-term follow-up when evaluating functional outcomes and should counsel patients regarding the expected timeline of recovery.

Several limitations of this study should be acknowledged. First, the number of included studies was relatively small, and the absence of randomized controlled trials limits the strength of the evidence. Second, most studies were conducted in East Asia, which may limit the generalizability of the findings. Third, variability in measurement methods (e.g., NAT vs. AI) and surgical techniques such as internal limiting membrane peeling, differences in PPV gauge size and baseline severity of aniseikonia, and the inclusion of combined procedures such as cataract surgery may have contributed to heterogeneity and may significantly influence visual perception outcomes. Finally, anisometropia is a major confounding factor in aniseikonia assessment, yet control for anisometropia was inconsistent across the included studies and may have influenced the reliability and comparability of the pooled estimates. These factors should be considered when interpreting the pooled estimates.

Sensitivity analyses were performed to address some of these concerns, including stratification based on anisometropia control and measurement methods (Figure 7B and Supplementary Figure S4). Although the overall direction of the findings remained broadly similar, methodological differences—including variability in measurement methods, surgical techniques, and anisometropia control—likely contributed substantially to inter-study heterogeneity and warrant careful consideration when interpreting the pooled estimates.

Future studies with larger sample sizes and standardized methodologies are needed to better elucidate the mechanisms underlying aniseikonia improvement and to identify reliable predictors of postoperative outcomes. In particular, further research is needed to clarify the relationship between macular microstructural changes and aniseikonia, which remains poorly understood.

## Figures and Tables

**Table 1:** Characteristics of included studies.

First Author	Year	Country	Study Design	No. of Eyes	F/U Duration (mo)	Method	Conclusion	PPV (Gauge)	Macular Config.	ROB
Han [13]	2016	KOR	Pros	24	6	AI	Sig.	25	N/A	Low
Hirata [14]	2019	JPN	Pros	30	12	NAT	Sig.	25	CMT, FAZ	Low
Khanna [15]	2022	FRA	Pros	42	24	NAT	Sig.	25	CMT, EZD	Low
Kim [16]	2021	KOR	Pros	84	6	NAT	Insig.	23 or 25	CMT, FAZ, EIFL	Low
Kim [9]	2022	KOR	Retro	79	6	NAT	Sig.	23	CMT, EIFL	Unclear
Kitagawa [17]	2019	JPN	Retro	13	6	NAT	Insig.	27	CMT, FAZ	Unclear
Moon [18]	2020	KOR	Retro	32	12	NAT	Insig.	23	CMT, INL, GCIPL	Unclear
Okamoto [19]	2014	JPN	Pros	44	6	NAT	Insig.	23 or 25	CMT, EZD, INL, GCIPL	Low
Okamoto [20]	2020	JPN	Pros	63	6	NAT	Insig.	N/A	N/A	Unclear
Takabatake [21]	2018	JPN	Pros	45	12	NAT	Sig.	N/A	CMT, EZD, INL, GCIPL	Unclear

No., number; mo, months; Conclusion, conclusion of aniseikonia changes; PPV, pars plana vitrectomy; Macular Config., Macular Configuration; ROB, risk of bias; KOR, Korea; JPN, Japan; FRA, France; Retro, retrospective; Pros, prospective; AI, Aniseikonia Inspector; NAT, the New Aniseikonia Test; Insig., insignificant; Sig., significant; N/A, not available; CMT, central macular thickness; FAZ, foveal avascular zone; EZD, ellipsoid zone disruption; EIFL, ectopic inner foveal layer; INL, inner nuclear layer; GCIPL, ganglion cell-inner plexiform layer.

**Table 2:** Correlation analyses between aniseikonia changes and changes or preoperative value of best-corrected visual acuity and central macular thickness in included studies.

	Δ Aniseikonia	
	Correlation *	*p*-Value *
Δ BCVA	0.6371	0.065
Δ CMT	0.0599	0.888
pre-BCVA	−0.3395	0.3373
pre-CMT	−0.3234	0.4346

* Spearman’s rank correlation coefficient. Δ, change between postoperative and preoperative values; BCVA, best-corrected visual acuity; CMT, central macular thickness; pre-, preoperative.

## Data Availability

All data generated or analyzed during this study are included in this published article.

## Supplementary Materials

The following supporting information can be downloaded at: https://www.mdpi.com/article/10.3390/jcm15114170/s1, Figure S1: Forest plots of changes in the best-corrected visual acuity (BCVA) at the final follow-up after pars plana vitrectomy (PPV) with subgroup analyses (A) for the study design and (B) for the follow-up period; Figure S2: Forest plots of changes in the central macular thickness (CMT) at the final follow-up after pars plana vitrectomy (PPV) with subgroup analyses (A) for the study design and (B) for the follow-up period; Figure S3. Forest plots of changes in macular microstructural parameters at the final follow-up after pars plana vitrectomy (PPV): (A) foveal avascular zone (FAZ), (B) inner nuclear layer (INL), (C) ganglion cell–inner plexiform layer (GCIPL), and (D) ellipsoid zone disruption (EZD). Analyses were performed using a random-effects model based on the inverse variance method for continuous outcomes (A–C) and the Mantel–Haenszel method for dichotomous outcomes (D). Figure S4. Sensitivity and subgroup analysis assessing the impact of measurement method and anisometropia control on changes in aniseikonia at the final follow-up after pars plana vitrectomy (PPV). The analysis excludes the study using the Aniseikonia Inspector and stratifies the remaining studies based on whether anisometropia was considered in the inclusion criteria; Table S1: The PRISMA 2020 checklist [26].
